# *Trypanosoma brucei rhodesiense* Transmitted by a Single Tsetse Fly Bite in Vervet Monkeys as a Model of Human African Trypanosomiasis

**DOI:** 10.1371/journal.pntd.0000238

**Published:** 2008-05-14

**Authors:** John K. Thuita, John M. Kagira, David Mwangangi, Enock Matovu, C. M. R. Turner, Daniel Masiga

**Affiliations:** 1 Trypanosomiasis Research Centre, (KARI-TRC), Kikuyu, Kenya; 2 Faculty of Veterinary Medicine, Makerere University, Kampala, Uganda; 3 Division of Infection and Immunity, Institute of Biomedical and Life Sciences, and Wellcome Centre for Molecular Parasitology, University of Glasgow, Glasgow, United Kingdom; 4 Molecular Biology and Biotechnology Department, International Centre of Insect Physiology and Ecology, Nairobi, Kenya; 5 Department of Biochemistry and Biotechnology, Kenyatta University, Nairobi, Kenya; Yale University School of Medicine, United States of America

## Abstract

We have investigated the pathogenicity of tsetse (*Glossina pallidipes*)-transmitted cloned strains of *Trypanosoma brucei rhodesiense* in vervet monkeys. Tsetse flies were confirmed to have mature trypanosome infections by xenodiagnosis, after which nine monkeys were infected via the bite of a single infected fly. Chancres developed in five of the nine (55.6%) monkeys within 4 to 8 days post infection (dpi). All nine individuals were successfully infected, with a median pre-patent period of 4 (range = 4–10) days, indicating that trypanosomes migrated from the site of fly bite to the systemic circulation rapidly and independently of the development of the chancre. The time lag to detection of parasites in cerebrospinal fluid (CSF) was a median 16 (range = 8–40) days, marking the onset of central nervous system (CNS, late) stage disease. Subsequently, CSF white cell numbers increased above the pre-infection median count of 2 (range = 0–9) cells/µl, with a positive linear association between their numbers and that of CSF trypanosomes. Haematological changes showed that the monkeys experienced an early microcytic-hypochromic anaemia and severe progressive thrombocytopaenia. Despite a 3-fold increase in granulocyte numbers by 4 dpi, leucopaenia occurred early (8 dpi) in the monkey infection, determined mainly by reductions in lymphocyte numbers. Terminally, leucocytosis was observed in three of nine (33%) individuals. The duration of infection was a median of 68 (range = 22–120) days. Strain and individual differences were observed in the severity of the clinical and clinical pathology findings, with two strains (KETRI 3741 and 3801) producing a more acute disease than the other two (KETRI 3804 and 3928). The study shows that the fly-transmitted model accurately mimics the human disease and is therefore a suitable gateway to understanding human African trypanosomiasis (HAT; sleeping sickness).

## Author Summary

Sleeping sickness is caused by a species of trypanosome blood parasite that is transmitted by tsetse flies. To understand better how infection with this parasite leads to disease, we provide here the most detailed description yet of the course of infection and disease onset in vervet monkeys. One infected tsetse fly was allowed to feed on each host individual, and in all cases infections were successful. The characteristics of infection and disease were similar in all hosts, but the rate of progression varied considerably. Parasites were first detected in the blood 4–10 days after infection, showing that migration of parasites from the site of fly bite was very rapid. Anaemia was a key feature of disease, with a reduction in the numbers and average size of red blood cells and associated decline in numbers of platelets and white blood cells. One to six weeks after infection, parasites were observed in the cerebrospinal fluid (CSF), indicating that they had moved from the blood into the brain; this was associated with a white cell infiltration. This study shows that fly-transmitted infection in vervets accurately mimics human disease and provides a robust model to understand better how sleeping sickness develops.

## Introduction

In human African trypanosomiasis (HAT), the use of animal models has contributed enormously to what is currently known about the relationships between disease duration, parasite invasion of different body systems and the potential of resultant host clinical and biological changes as diagnostic and disease staging markers. Several host-parasite model systems have been developed, based on infection of various hosts with the livestock pathogen *Trypanosoma brucei brucei* and to a lesser extent the human pathogens *T. b. rhodesiense* and *T. b. gambiense*. Characterisation of these HAT models shows that the disease occurs in two stages irrespective of host: an early haemo-lymphatic trypanosome proliferation, and a late central nervous system (CNS) infection, indicating that the basic pattern is similar to the disease in humans. This is evidenced by demonstration of trypanosomes, first in the haemo-lymphatic system and later in the CNS of the mouse model with subsequent cerebral pathology [1],[2]. Models based on larger mammals such as the chimpanzee *T. b. rhodesiense* model [3], the vervet monkey *T. b. rhodesiense* model [4] and the sheep *T. b. brucei* model [5], also follow a similar two-stage disease pattern. These, unlike rodents, allow collection of cerebrospinal fluid (CSF) that has been used to demonstrate elevation of white cell counts and total protein levels as indicators of CNS stage disease [6].

The KETRI vervet monkey model has been reported to closely mimic HAT clinically, immunologically and pathologically [4], [7]–[9]. However, these previous studies were limited in scope in three important ways. Firstly, infections were initiated by intravenous inoculation (syringe) of bloodstream form trypanosomes as opposed to the natural human disease, which begins via the bite of a tsetse fly, with the intra-dermal inoculation of metacyclic trypanosomes. The difference between the two routes of infection has the potential to affect trypanosome virulence and subsequent disease pathogenesis that has been little explored to date. Second, disease progression has been monitored mainly in terms of clinical symptoms, gross pathology, histo-pathology and antibody responses [4],[7], with little reference to the development of blood pathology. Third, only a single strain of trypanosomes, KETRI 2537 [9], has been adequately characterised even though trypanosome strains vary in the severity of pathogenesis and virulence [10]–[11].

The present study was designed to address these limitations and thus improve further the potential utility of the model and our understanding of pathogenesis in trypanosome infections. We characterised the pathogenicity of *T. b. rhodesiense* in vervet monkeys, following infection from the bite of a single tsetse fly, hence mimicking the natural route of infection in man. This allowed us to measure a range of parameters in blood and cerebrospinal fluid (CSF) including several that had not previously been studied. It was thus possible to measure the development of clinical complications of HAT infections, such as anaemia, more precisely. We describe the development of clinical pathology resulting from infection with four cloned strains of *T. b. rhodesiense*.

## Materials and Methods

### Ethics

This study was undertaken in adherence to experimental guidelines and procedures approved by the Institutional Animal Care and Use Committee (IACUC), the ethical review committee for the use of laboratory animals.

### Selection of stock trypanosome isolates and cloning

Trypanosome isolates that were used in this study (Table 1) were all initially obtained through collection of infected blood from patients in the western Kenya/eastern Uganda focus of endemic *T. b. rhodesiense* sleeping sickness (historically known as the Busoga focus). All the isolates are maintained as cryo-preserved stabilates in the KARI-TRC (formerly KETRI) trypanosome bank. The isolates were included in the study on the basis of the year of isolation, to give a wide temporal distribution and the locality of isolation to give a wide spatial distribution within this sleeping sickness focus. The selected stabilates were cloned using the hanging drop method described by Herbert and Lumsden [12].

**Table 1:** Temporal and geographic distribution of *T. b. rhodesiense* isolates and clones.

Year of isolation	geographic location	Isolate lab. No.	Comments	Lab No. of derivative clone
1972	Busoga, Uganda	KETRI 2537	Used in Schmidt and Sayer, (1982); five passagessince isolation, well characterised.	KETRI 3741
1989	Busia, Kenya	KETRI 3199	One passage since isolation	KETRI 3801
1989	Bukhayo West, Kenya	KETRI 3205	Two passages since isolation	KETRI 3804
2003	Tororo, Uganda	KETRI 3928	One passage since isolation	KETRI 3928

### Tsetse fly infections

Male teneral tsetse flies (*Glossina pallidipes*) were obtained from the KETRI colony initially established with pupae from the Lambwe Valley of Kenya, which is part of the western Kenya/eastern Uganda focus of HAT. In order to initiate infection of tsetse flies with each trypanosome clone, four sub-lethally irradiated (600 rads, 5 minutes) donor Swiss White mice were each inoculated intraperitoneally with 0.2 millilitres of the thawed *T. b. rhodesiense* stabilates, diluted in phosphate saline glucose (PSG). At peak parasitaemia, typically approximately 10^8^ trypanosomes per millilitre, a batch of 50 teneral flies were allowed to feed essentially as described [13], and maintained thereafter on clean bovine blood by feeding via a silicon membrane. Thirty days after the infective blood meal, all the flies were chilled briefly and separated into individual fly cages. The flies with mature trypanosome infections were then identified by xenodiagnosis using Swiss White mouse. We were repeatedly unable to find trypanosomes in salivary probes on warm microscope slides [14].

### Experimental monkeys

Nine vervet monkeys (*Chlorocebus aethiops*, African Green Monkeys) of both sexes weighing between 2.7 and 5.2 kg were acquired from the Institute of Primate Research (IPR) in Kenya. They were housed in quarantine for a minimum of 90 days while being screened for evidence of disease, including zoonoses as described by Ndung'u and colleagues [8]. They were also dewormed and treated for any ectoparasite infestations. During the quarantine period, the animals became accustomed to staying in individual squeeze-back stainless steel cages and human handling.

During quarantine and also while in the experimental animal wards, the monkeys were maintained on green maize, fresh vegetables (bananas, tomatoes and carrots) and commercial monkey cubes (Monkey cubes, Unga Ltd, Kenya), fed twice daily (9.00–9.30 am and 3.00–3.30 pm), and given water *ad libitum.* After the expiry of the 90 days quarantine, the study animals were then transferred to experimental wards and acclimatised for a further two weeks prior to commencement of pre-infection data collection.

### Infections in monkeys

The monkeys were randomly allocated into four experimental groups, each containing at least one male and one female, for infection with *T. b. rhodesiense* clones as follows: KETRI 3741 (three monkeys, #s. 476, 515, and 536), KETRI 3801 (monkey #s. 523, 579), KETRI 3804 (monkey #s 556 and 574) and KETRI 3928 (monkeys #s 554 and 555). Pre-infection (baseline) data was collected over a period of 14 days after which each monkey was infected by allowing one tsetse fly, confirmed trypanosome positive through mouse infectivity tests, to feed on a shaved part of its thigh, while the monkey was under ketamine Hcl (Rotexmedica, Trittau Germany) anaesthesia. Before and following the infective tsetse bite, the monkeys were monitored for activity, posture, demeanour and general clinical presentation on a daily basis. Appetite was assessed daily, by scoring the proportion of the daily feed ration consumed by each monkey on a scale of 0 (no food eaten), 1/4, 1/2, 3/4 and 1 (full ration eaten).

Parasitaemia was assessed daily using the method of Herbert and Lumsden [12], using heparinised capillary blood drawn from the ear vein, starting from the third day after infection. Every four days, the monkeys were sedated using ketamine hydrochloride (10–15 mg per kg body weight intramuscularly) after which a detailed clinical examination was carried out and 2 ml of venous blood (femoral) sampled for a full haemogram. Every eight days, a CSF sample was also collected through lumbar puncture for assessment of CNS parasitosis and white cell numbers. The experiment was terminated through humane euthanasia at *extremis*. An individual animal was judged to be *in extremis* when for three consecutive days it was either unable or reluctant to perch, had very low feed intake (<1/4 of daily ration), and in addition had signs of advanced late stage disease (e.g somnolence). Euthanasia was carried out using 20% pentobarbitone sodium (Euthatal, Rhone Merieux).

### Sample analysis

Cerebrospinal fluid white cell counts (WCC) and total trypanosome numbers were concurrently counted using a Neubert chamber as previously described [6],[8]. Immediately after every sampling session (not exceeding one hour), total red blood cell (RBC) and related indices, white cell numbers and differential, platelet (thrombocyte) counts and associated parameters were determined using an AC^3diff^ T Coulter counter (Miami, Florida, USA).

### Data analysis

Data was entered and managed using Microsoft Excel (Version 2003). Statistical analysis was conducted using Statview for Windows Version 5.0.1 (SAS Institute Inc, 1995–1998, Cary, NC). The behaviour of the four trypanosome strains was analysed and is presented as tables and or graphs representing time bound changes in individual infected monkeys' clinical, haematological and cerebrospinal fluid pathology data. In addition, descriptive statistics [mean (and the corresponding 95% confidence intervals, CI), or medians and range] were derived for the entire group of nine monkeys. In addition to derivation of descriptive data, haematology data was further analysed using repeated measures ANOVA. Finally, Spearman's correlation coefficients were determined to assess the strength of association between CSF trypanosome and white cell numbers.

## Results

### Progression of infection in vervet monkeys

#### Parasitaemia

**Figure 1:**
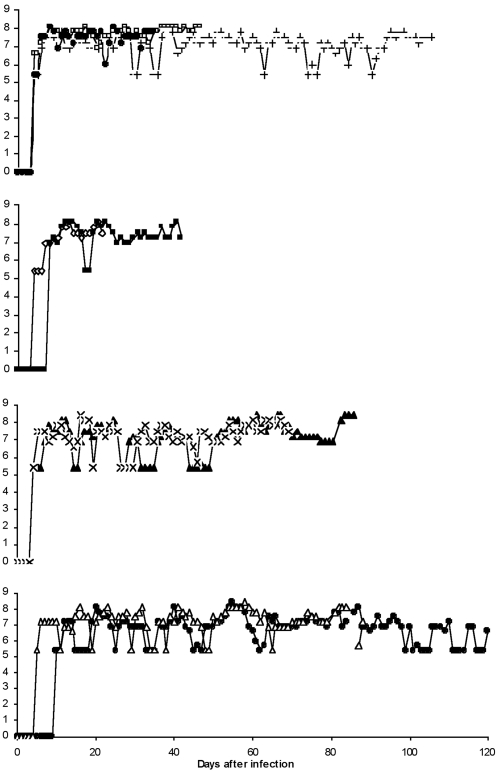
Parasitaemia curves generated by four *T.b. rhodesiense* cloned strains after experimental fly (*Glossina pallidipes)* infection of nine vervet monkeys (+-476, □-515, ♦-536, ◊-523, ▪-579, ▴-556, x-574, •-554, ▵-555). After a variable pre-patent period, the parasitaemia tended to plateau but with more clearly defined waves of relapse and recrudescence in the individuals with longer disease duration.

Following the bite of a single infected tsetse fly, parasitaemia was detected in the peripheral blood of all nine monkeys (Table 2), with an overall median pre-patent period of 4 (range = 4–10) days. The parasitaemia caused by all strains increased rapidly (Figure 1), attaining peak levels of antilog 8.1–8.4 trypanosomes/millilitre of blood within three to four days in different individuals. Although there were subsequent fluctuations (2–3 log scales), the parasitaemia levels remained high to very high throughout the infection for all animals/strains (Figure 1). However, slight differences were noted in the mean daily parasitaemias for different individuals, with monkey 554 (strain KETRI 3928) having lowest mean levels (antilog 6.8).

**Table 2:** Evolution of clinical parameters of vervet monkeys that were infected with *T. b. rhodesiense* cloned strains through tsetse (*Glossina pallidipes*) transmission.

Vervet monkey clinical data		T.b.r. KETRI 3741			T.b.r. KETRI 3801		T.b.r. KETRI 3804		T.b.r. KETRI 3928	
		^a^476F	^a^515M	^a^536M	^a^523F	^a^579M	^a^556F	^a^574M	^a^554F	^a^555M
Appearance of trypanosome chancre: dpi when first seen		NO	4	4	NO	NO	4	4	NO	4
Pre-patent period: dpi		4	4	4	4	8	4	4	10	5
Enlargement of superficial lymph nodes: dpi when first seen		8	8	12	8	8	8	8	12	4
Splenomegaly	dpi when first seen	12	8	12	8	12	8	32	16	32
	Size relative to pre-infection	×1.5	×1.5	×2	×1.5	×1.5	×1.5	×1.5	×2	×1.5
Muscle tremors: dpi when first seen		NO	NO	NO	NO	12	NO	NO	NO	NO
Cerebrospinal Fluid parasitosis	First detection: dpi	16	24	16	16	8	16	24	40	16
	No. of trypanosomes in CSF during the entire infection: median (range)	1 (0–123)	15.5 (0–26)	2 (0–475)	1 (0–1)	1 (0–31)	4 (0–31)	1.5 (0–1620)	1 (0–10)	3.5 (0–363)
CSF white cell (WC) changes	Pre-infection: median (range)	1 (0–8)	6 (3–9)	7 (7–8)	0	1 (1–3)	4 (1–6)	1 (0–1)	3 (0–7)	1 (1)
	During infection: median (range)	16 (4–91)	11 (2–45)	14 (4–20)	3 (0–7)	9 (2–14)	8 (0–39)	7.5 (0–69)	4 (0–20)	17 (3–46)
Ataxia/incoordination: dpi when first seen		NO	NO	28	16	35	NO	NO	115	NO
Oedema of eyelids, vulva or scrotum: dpi when first seen		84	NO	NO	NO	37	76	NO	NO	NO
Abnormal posture		NO	NO	NO	O	O	NO	NO	NO	NO
Lethargy/reduced activity at extremis		O	NO	O	O	O	O	O	O	O
Somnolence at extremis		O	NO	O	O	O	NO	NO	O	NO
Inability or extreme reluctance to perch: dpi when first seen		104	46	34	NO	NO	NO	NO	NO	88
Duration of disease up to euthanasia at extremis (dpi)		104	46	34	21	41	84	68	120	88
Reduced appetite	Proportion of days in which feed intake was 50% or below of daily ration (pre-infection)	3/14 (21.4%)	0/14 (0%)	0/14 (0%)	1/14 (7.1%)	0/14 (0%)	0/14 (0%)	0/14 (0%)	0/14 (0%)	0/14 (0%)
	Proportion of days in which feed intake was 50% or less of daily ration (after infection)	13/104 (12.5%)	5/46 (10.9%)	12/34 (35.3%)	11/21 (52.4%)	2/41 (4.9%)	0/84 (0%)	1/68 (1.5%)	0/120 (0%)	1/120 (0.8%)
Pre-infection weight (kg)		2.7	3.4	4.1	2.7	4.3	2.9	4.0	3.6	5.2
%weight loss at 32 dpi		3.8	10.3	32.1	NDA	23.3	7.0	13.8	15.5	12.6

Key: dpi = days post infection; T.b.r. = *Trypanosoma brucei rhodesiense*; a = Laboratory number of monkey; F = Female; M = Male; O = observed; NO = Not observed; − = decreased; + = increased.

#### Clinical symptoms

Clinical symptoms of early stage HAT included the classical trypanosome chancres (swellings 2–5 cm in diameter with circumscribed erythematous margins), which developed in five of the nine (55.6%) individuals (Table 2). These swellings were detected by the fourth day and lasted up to eight days post infection (dpi). Superficial lymph nodes (axillar and inguinal) and splenomegally were enlarged in all monkeys, starting 4–12 dpi and 8–32 dpi respectively (Table 2). Raised hair coats, lethargy, progressive weakness, intermittent fever and varying degrees of weight loss were similarly observed in all individuals. Anorexia was most marked in vervet monkeys infected with strains KETRI 3741 and KETRI 3801. In these monkeys daily feed intake reduced in comparison with the pre-infection period; in vervet 523 feed intake was below 50% of daily ration in 11/21 (52.4%) days. Other clinical signs included muscle tremors/fasciculation, seen only in monkey 579 (KETRI 3801) and superficial oedema in 3/9 of the monkeys. Ataxia (inco-ordination), hind leg paralysis and somnolence were observed in a few individuals at extremis (Table 3), suggesting that the nervous system was affected. The monkeys were euthanized humanely at extremis with a median duration of infection of 68 (range = 21–120) days.

**Table 3:** Progressive changes in haematology indices of vervet monkeys infected with *T.b. rhodesiense* cloned strains through cyclic (tsetse; *Glossina pallidipes*) transmission.

Parameters	*T.b. rhodesiense* KETRI 3741			*T.b. rhodesiense* KETRI 3801		*T.b. rhodesiense*. KETRI 3804		*T.b.rhodesiense* KETRI 3928	
	^a^476F	^a^515M	^a^536M	^a^523F	^a^579M	^a^556F	^a^574M	^a^554F	^a^555M
Red blood cells (countsx10^6^/µl): pre-infection level	5.21	5.71	6.35	6.16	6.28	5.48	6.51	5.39	7.34
Red blood cells: % reduction at 32dpi	23.4	25.7	53.5	48.9	44.4	29.7	26.7	21.3	24.8
Red blood cells: % reduction at extremis	55.9	15.4	53.5		49.0	71.4	52.2	13.2	57.9
Haematocrit (%): pre-infection level	40.6	46.6	51.4	48.3	48.3	40.2	52.1	41.7	57.2
Haematocrit (%): % reduction at 32 dpi	33.3	33.3	59.5	47.8	45.8	30.3	35.1	26.9	32.9
Haematocrit (%): % reduction at extremis	56.2	31.1	55.1		50.0	68.7	59.9	24.5	63.1
Haemoglobin (g/dl): Pre-infection level	12.2	14.3	16.4	14.2	14.2	11.8	15.2	12.1	16.9
Haemoglobin: % reduction at 32 dpi	25.4	31.5	57.3	45.8	45.1	30.5	34.9	24.8	32.0
Haemoglobin: % reduction at extremis	58.2	21.7	57.9		47.9	67.8	59.2	17.4	62.1
Mean corpuscular volume (fl): Pre-infection level	77.8	81.6	80.9	78.4	76.8	73.5	79.9	77.4	77.9
Mean corpuscular volume: % change at 32 dpi	−12.6	−9.9	−12.6	+2.0	−2.5	−1.1	−11.1	−6.8	−0.7
Mean corpuscular volume: % change at extremis	−0.9	−11.3	+1.1		−1.6	+9.5	−15.6	−12.9	−12.5
Mean corpuscular haemoglobin (pg): Pre-infection level	23.4	25.1	25.6	23.1	22.6	21.6	23.3	22.4	23.1
Mean corpuscular haemoglobin: % change at 32 dpi	−3.0	−8.0	−7.8	+6.5	−0.4	−1.4	−10.3	−4.0	−10.0
Mean corpuscular haemoglobin: % change at extremis	−5.6	−7.6	−5.8		+2.7	+13.0	−13.7	−4.9	−9.5
Red cell distribution width (%): pre-infection level	13.4	13.7	11.8	14.8	13.6	13.5	11.9	12.4	12.6
Red cell distribution width: % increase at 32 dpi	29.9	56.9	61.9	17.6	61.0	57.0	46.2	46.8	34.9
Red cell distribution width: % increase at extremis	56.7	36.5	57.6		77.9	79.3	68.9	93.5	50.0
Platelets (counts/µl): Pre-infection level	461	281	307	330	331	457	266	374	341
Platelets: % reduction at 32 dpi	38.8	7.7	71.7	99.1	77.6	78.1	68.8	53.5	76.2
Platelets: % reduction at extremis	76.4	30.0	78.5		82.8	98.0	89.1	97.9	94.4
Mean platelet volume (fl): pre-infection values	7	6.3	6.9	7	7.7	7.6	6.7	7.3	6.3
Mean platelet volume: % increase at 32 dpi	17.1	23.8	50.7	87.4	53.2	NDA	55.3	17.1	54
Mean platelet volume : % increase at extremis	45.7	47.6	60.9		49.4	55.3	NDA	41.1	85.7

Key: dpi = days post infection; T.b.r. = *Trypanosoma brucei rhodesiense*; a = Laboratory number of monkey; F = Female; M = Male; O = observed; NO = Not observed; fl = femtolitres; pg = picograms; g/dl = grams per decilitre; − = decreased; + = increased; NDA = No data available.

#### Haematology

Anaemia was a key feature of the cyclic infection in monkeys. During the first 32 dpi, red blood cell (erythrocyte) counts and associated indices declined rapidly; the magnitude of the decline in these parameters was most pronounced in three monkeys infected with strains KETRI 3801 (523, 579) and KETRI 3741 (No 536) as shown (Table 3). In the same period, the mean red blood cell count declined by 32.8%, from a pre-infection value of 6.1 (95% CI = 5.5–6.6) to 4.1 (95% CI = 3.5–4.8; p<0.0001) million cells/µl of blood. The red cell associated parameters exhibited similar trends, with the haematocrit (HCT) declining from a pre-infection mean value of 47.4 (95% CI = 42.9–51.8) to 29.5 (95% CI = 25.1–33.9, p<0.0001). Similarly, the haemoglobin concentration declined from a pre-infection mean value of 14.1 (95% CI = 12.7–15.6) to 9.1 (95% CI = 7.8–10.2) grams/decilitre (g/dl) of blood. As the infection progressed, erythrocyte numbers and associated parameters stabilised and actually improved slightly in two monkeys (515 and 554, Table 3). At the terminal stage when the monkeys were euthanized, the overall mean HCT was 23.5 (95% CI = 18.3–28.7, p<0.0001) while the mean haemoglobin content was 7.2 (95% CI = 5.4–9.0) g/dl.

**Figure 2:**
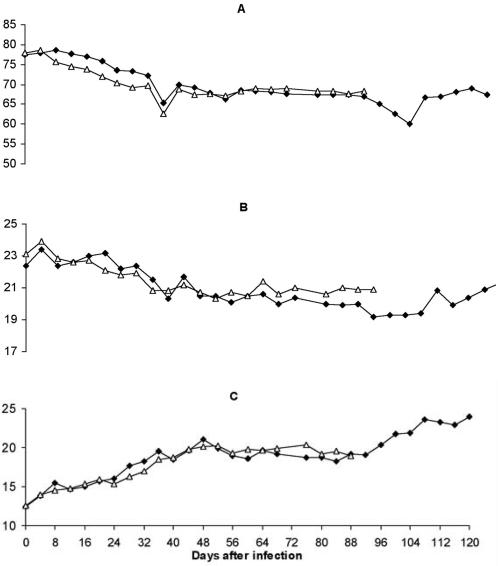
Changes in the mean corpuscular volume (MCV) mean corpuscular haemoglobin (MCH) and Red cell distribution width (RDW) in two vervet monkeys (•-554 and ▵-555) that were infected with *T.b. rhodesiense* KETRI 3928. Panel A: MCV; Panel B: MCH; and Panel C: RDW. Note that while both MCV and MCH decreased below pre-infection levels and remained low throughout; RDW increased throughout the infection.

**Figure 3:**
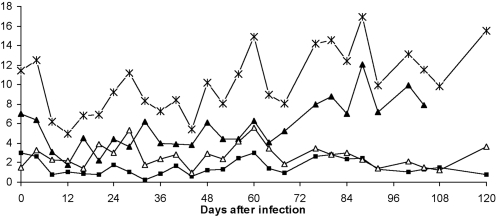
Changes in total and differential white blood cell numbers in vervet 554 that was infected with *T.b. rhodesiense* KETRI 3928. During the early part of the infection, granulocytes and lymphocytes are approximately equal while later in the infection, lymphocytes are clearly the predominant cell type. (X- Total WBC, ▴-lymphocytes, ▵-Granulocytes, ▪-Monocytes).

The resultant anaemia was characterised by an overall reduction in the average size of the red cells (the mean corpuscular volume, MCV) and the mean corpuscular haemoglobin (MCH); these changes were in contrast to the red cell distribution width (RDW) which increased throughout the infection (Table 3, Figure 2). Overall, the mean MCV declined significantly (p = 0.0001), from a pre-infection (0 dpi) value of 78.2 (95% CI = 76.4–80.1) to 71.6 (95% CI = 69.7–73.4) by 32 dpi. The mean MCH declined more slowly; it took 52 days to decline significantly (p = 0.007) from a pre-infection value of 23.4 (95% CI = 22.4–24.4) to 20.6 (95% CI = 19.9–21.4) g/dl. Meanwhile, the mean RDW increased significantly (p = 0.0001) from a pre-infection (0 dpi) value of 13.1 (95% CI = 12.3–13.8) to 19.2 (95% CI = 17.5–20.9) by 32 dpi. Considered together, changes in MCV, MCH and RDW indicate that: i) the monkeys suffered microcytic hypochromic anaemia ii) the heterogenicity (anisocytosis) of the erythrocyte population increased throughout the infection. In most individuals, the MCV and MCH stabilised and, in vervet 556, increased beyond pre-infection values in later stages of infection (Table 3, Figure 3). These changes coincided with stabilisation or improvement in erythrocyte numbers showing that in this small number of individuals, the anaemia became regenerative.

The experimental cyclic disease was further characterised by a marked thrombocytopenia. The pre-infection mean platelet count was 350.4 (95% CI = 297.4–403.5) platelets/µl of blood. Following infection, platelets declined rapidly and progressively, reaching a mean level of 161.6 (95% CI = 94.3–228.8) platelets/µl of blood by 8dpi, which was a 53.9% drop. The sudden decrease in platelet numbers was countered by a slightly delayed but similarly dramatic increase in mean platelet volume (MPV). From a pre-infection mean volume of 7.0 (95% CI = 6.6–7.4) fl, the MPV increased by 25.7% to a mean volume of 8.8 (95% CI = 7.2–10.4) fl by 12 dpi and had increased further to 9.7 (8.3–11.0), a 38.6 % increase by 32 dpi.

Early in the infection, the white blood cell (WBC) response involved contrasting changes in the numbers of granulocytes and lymphocytes in comparison with the pre-infection values. At 4 dpi, granulocyte counts increased in all monkeys (except vervet 476) as shown (Table 4). The mean granulocyte count rose three-fold, from a pre-infection (0 dpi) mean value of 1.2 (95% CI = 1.0–1.4) to a mean value of 3.7 (95% CI = 1.5–6.0; p = 0.0002) cells/µl of blood. In contrast, lymphocyte counts declined rapidly over the same period (Table 4) and continued to be low well into the second week of the infection. At 8 dpi, the mean count had declined by 57.5% from a pre-infection (0 dpi) value of 4.0 (95% CI = 2.8–5.2)×10^3^ to a mean value of 1.7 (95% CI = 0.9–2.5)×10^3^ cells/µl of blood at 8 dpi. This decline was significant (p = 0.0001). Monocyte numbers decreased less convincingly, from a pre-infection mean value of 1.3 (95% CI = 0.7–2.0) to a mean value of 0.7 (95% CI = 0.5–0.9)×10^3^ cells/µl of blood at 8 dpi (p = 0.03). All these changes resulted into an overall leucopaenia between 4–12 dpi, the same period that trypanosomes were first detected in peripheral blood in different monkeys. After the initial leucopaenia, multiple peaks of WBC were observed in all monkeys as shown for vervet 554 (Figure 3). In very late stage infections, overall leukocyte densities increased above pre-infection values (leucocytosis) in 3/9 (33%) monkeys, largely determined by changes in lymphocyte numbers (Table 4; Figure 3). In vervet 579 (KETRI 3801) however, granulocytes remained elevated above pre-infection levels (and actually higher than lymphocyte numbers) throughout the infection.

**Table 4:** Blood white cell changes in vervet monkeys infected with *T.b. rhodesiense* cloned strains through cyclic (tsetse; *Glossina pallidipes*) transmission.

Parameters		*T.b. rhodesiense* KETRI 3741			*T.b. rhodesiense* KETRI 3801		*T.b. rhodesiense*. KETRI 3804			
		^a^476F	^a^515M	^a^536M	^a^523F	^a^579M	^a^556F	^a^574M	^a^554F	^a^555M
Total white cell counts (×10^3^ )/µl of blood	0 dpi	8	3.4	4.4	5.8	6.6	7.4	6	11.4	5.4
	4 dpi	5.2	4.1	4.7	8.4	5.9	12.2	NDA	12.5	6.2
	8 dpi	5.5	1.8	3.2	2.6	4.6	4.8	4	6.2	5.1
	12 dpi	5.8	2.7	2.8	NDA	4.4	8.4	6.3	5	4.3
	32 dpi	5.4	4.8	4.4	NDA	NDA	8.1	8.2	8.3	NDA
	terminal	3.9	3.4	3.9	2.9	3.8	16.6	3.6	15.5	4.8
Lymphocytes counts (×10^3^)/µl of blood	0 dpi	5.6 (70)	2.2 (65)	2.4 (55)	4.2 (72)	4 (61)	4.6 (62)	3 (50)	7 (61)	2.9 (54)
	4 dpi	3.7^b^ (71)	0.8^b^ (20)	1.2^b^ (26)	5.1^b^ (61)	1.9 (32)	1.8^b^ (15)	NDA	6.4 (51)	1.7^b^ (27)
	8 dpi	3.7 (67)	0.7 (39)	1.5 (47)	1 (39)	0.6^b^ (13)	1.8^b^ (38)	1.7 (43)	3.1^b^ (50)	1.3^b^ (26)
	12 dpi	4 (69)	1.3 (48)	1.2 (43)	NDA	1.2 (27)	3.7 (44)	3.2 (51)	1.8^b^ (36)	1.7 (40)
	32 dpi	2.7 (50)	1.7 (35)	2.6 (60)	NDA	NDA	5.3 (65)	4.7 (57)	6.2 (75)	NDA
	Terminal	2.1 (54)	2.1 (62)	2.1 (53)	1.4 (48)	1.7 (45)	8 (48)	1.6 (44)	12.6 (81)	3 (63)
Monocytes counts (×10^3^)/µl of blood	0 dpi	1.1 (14)	0.3 (9)	1 (22)	0.5 (9)	1.6 (24)	1.6 (22)	1.5 (25)	3 (26)	1.5 (28)
	4 dpi	1 (19)	0.3 (7)	0.5 (11)	0.9 (11)	0.4 (7)	0.5 (4)	NDA	2.7 (22)	0.2 (3)
	8 dpi	0.4 (7)	0.4 (22)	1 (31)	0.3 (12)	0.5 (11)	0.8 (17)	0.8 (20)	0.8 (13)	0.9 (18)
	12 dpi	0.8 (14)	0.4 (15)	0.3 (10)	NDA	0.5 (11)	0.3 (4)	1 (16)	1.1 (22)	0.8 (19)
	32 dpi	1.1 (20)	1 (21)	1 (24)	NDA	NDA	1.1 (14)	1.4 (17)	0.3 (4)	NDA
	Terminal	0.7 (18)	0.2 (5)	1.2 (31)	0.2 (7)	1.1 (29)	3.9 (24)	1.1 (31)	0.8 (5)	0.7 (15)
Granulocytes counts (×10^3^)/µl of blood	0 dpi	1.3 (16)	0.8 (25)	1.1 (24)	1.1 (19)	1 (15)	1.2 (16)	1.5 (25)	1.5 (13)	1.1 (20)
	4 dpi	0.6 (12)	3 (74)	3 (65)	2.4 (29)	3.6 (61)	9.8 (80)	NDA	3.3 (26)	4.2 (68)
	8 dpi	1.4 (26)	0.7 (38)	0.7 (23)	1.3 (50)	3.6 (78)	2.3 (48)	1.6 (40)	2.3 (37)	2.9 (57)
	12 dpi	1 (17)	1 (36)	1.3 (47)	NDA	2.7 (61)	4.3 (51)	2.1 (33)	2.2 (44)	1.8 (42)
	32 dpi	1.6 (30)	2.1 (44)	0.7 (16)	NDA	NDA	1.7 (21)	2.1 (26)	1.8 (22)	NDA
	Terminal	1.2 (31)	1.2 (34)	0.7 (17)	0.8 (28)	1 (26)	4.7 (28)	0.9 (25)	3.6 (23)	1.2 (25)

Key: dpi = days post infection; T.b.r. = *Trypanosoma brucei rhodesiense*; a = Laboratory number of monkey; b = value corresponding to the prepatent period; F = female; M = male; O = observed; NO = not observed; NDA = No data available; Numbers in parentheses: cell type expressed as a % of the total white cell count at each time point.

#### Cerebrospinal fluid (CSF) changes

**Figure 4:**
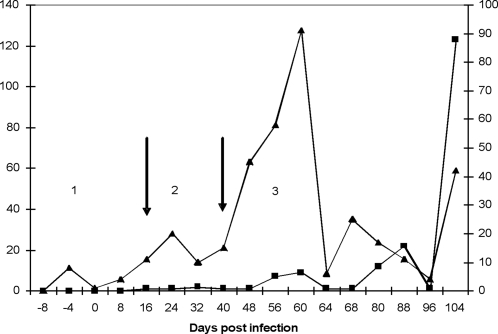
Changes in cerebrospinal fluid (CSF) trypanosomes (▪) and white cell (▴) during the course of infection of vervet 476 with *T.b. rhodesiense* KETRI 3741. Phase 1: early stage infection; Phase 2: transition of intermediate stage infection and phase 3: late stage disease which at terminal point was marked by surge in trypanosome numbers and white cell counts.

The period to penetration of parasites into the CSF varied between strains and individual monkeys, with a range of 8–40 (overall median 16) days (Table 2). Subsequently, parasite numbers and white cell counts (WCC) became elevated, the increase in magnitude becoming more severe as the infection progressed as shown for vervet 476 that was infected with clone KETRI 3741 (Figure 4). The changes depicted in the vervet infection show a distinct early stage phase that was characterised by localization of trypanosomes within the haemo-lymphatic system, with no trypanosomes or pathological changes in the CSF. Once the trypanosomes invaded the CSF, a transition or “lag phase” was evident, characterised by low trypanosome numbers in the CSF, with a median count of 1 [(range = 1–6); mean = 1.6 (95% CI = 0.3–2.8)] trypanosomes/µl and in vervet 476, lasted for about 14 days. Further WCC in CSF were also moderately elevated, with a median count of 8 [(range = 0–41); mean = 11 (95% CI = (1.6–20.4)] cells/µl, in comparison with a pre-infection median count of 2 [range = 0–9; mean = 2.6 (95% CI = 0.6–5.1) cells/µl. The third phase, a severe or true late stage disease phase was characterised by greatly elevated numbers of CSF trypanosomes culminating in terminal stage having a median count of 31 [range = 1–1620; mean = 296.7 (95% CI = 0–700.4)] trypanosomes/µl of CSF. At the same time the WCC were also further elevated with a median count of 14 [range = 7–46; mean = 23.7 (95% CI = 11.5–35.9) cells/ µl of CSF. Overall, there was a positive linear association between trypanosomes and WCC in the CSF (r_s_ = 0.3244, p = 0.001).

## Discussion

In this study, infection of vervet monkeys was initiated by the bite of a single infected tsetse fly. To our knowledge, this represents the first time single fly transmission of *T. b. rhodesiense* clones in vervet monkeys (or any other primate model) has been achieved, hence establishing a model that more accurately mimics the transmission of sleeping sickness as it occurs in humans. Information on natural HAT relies on data provided in case reports [15]–[16] and sometimes re-analysis of retrospective clinical, epidemiological and pathology data [17]–[18]. Such data are naturally limited on the questions of disease onset and duration as there are only a small number of case reports in which the patient could accurately remember the exact time of being bitten by a tsetse fly [16],[18]. In any case this is not readily accessible information for inhabitants of endemic areas, where tsetse fly challenge is continuous. This study has allowed us to generate information on the pathogenesis of HAT in a manner that more accurately mimics human disease, and facilitates documentation of data from precise sampling points during the course of a tsetse transmitted infection.

All four *T. b. rhodesiense* clones that were selected for this study were successfully transmitted through tsetse flies in the initial step of the model development protocol, a result that is consistent with the very good vectorial capacity of *G. pallidipes* for both human and animal trypanosomiasis in eastern Africa. Tsetse flies carrying mature infections were identified by xenodiagnosis using Swiss White mice. The pre-patent period in these mice (data not shown), and subsequently in monkeys (Table 2), showed considerable variation from host to host, consistent with observations in HAT patients [18]–[19]. In contrast, mice and vervet monkeys that are infected with *T. b. rhodesiense* via syringe passage show less variation in pre-patent period [4], presumably because the inoculum in these is usually well-defined, while tsetse flies are estimated to inject 0–40,000 (mean, 3,200) infective metacyclics [20].

Trypanosomes were detected in 7 of the monkeys within 5 days after infection (Table 2), indicating that the movement of trypanosomes from the site of the fly bite to the systemic circulation occurred quickly. This is remarkable, because it must be associated with the transition from non-proliferating metacyclics to rapidly dividing long slender bloodstream forms, clearly a survival strategy for the parasites. This movement was independent of the development of chancre, which was only observed in 5 monkeys. Chancres have been observed in HAT patients in whom these swellings are estimated to occur within 5–15 days of an infective fly bite [20], consistent with our data (Table 2). During the formation of the chancre, the metacyclics transform to rapidly dividing bloodstream form trypanosomes while the tissue at the inoculation site mounts a reaction characterized by a marked infiltration with polymorphonuclear leucocytes [21]–[22]. The immune reaction generated at the chancre is responsible for development of specific immunity against the variable antigen type of metacyclics [23].

The finding that the severity of the clinical disease differed between individual monkeys that were infected with the same strain emphasized the likely role of host immunity on disease outcome. The ability/inability of the host to control parasitaemia and its effect on disease duration is further indicated by the observation that parasitaemia patterns showed more fluctuation (clearly marked waves) in individuals with longer disease duration than in those with shorter durations (Figure 1). A similar trend was observed in mice (data not shown), consistent with previous reports [11]. In our study, clone 3801 produced a more acute disease, while clone 3928 manifested the most chronic disease; the other two clones were intermediate. These observations suggest that the parasites have intrinsic properties that, in part, determine virulence. Host factors also contribute to the disease profile, as evidence from variations in animals infected with the same clone indicates. A study of a number of isolates from eastern Uganda by Smith and Bailey [24] in mice showed that distinct acute and chronic strains of *T. b. rhodesiense* circulate in the focus and each strain is related to a given zymodeme. However, apart from individual parasite variations there were no features that could distinguish the Ugandan from Kenyan isolates. This supports the view that the four strains used in this study belong to the same endemic focus characterized by pockets of specific zymodemes with distinct clinical manifestations [24]. Similar diversities in clinical manifestations have been observed in HAT patients infected with *T. b. rhodesiense* [25] and *T.b. gambiense* [26] showing that animal models accurately mirror the situation in humans

Haematology results showed that anaemia developed early in the monkey infections; the decline in relevant parameters was detected as early as 8 dpi. However, the rate of decline of RBC and associated parameters was much slower than in *T. brucei* infected mice in which the numbers of circulating erythrocytes can fall by up to 50% within a week after infection [27]. Anaemia is a common occurrence in both *T. b. rhodesiense* and *T. b. gambiense* forms of sleeping sickness [15]–[16],[26],[28], similar to the case in vervet monkeys. However, determination of the rates of decline of RBC and associated parameters, is not possible in humans since neither the date of infection nor the pre-infection values in individual patients are known. The type of anaemia reported in our study, microcytic hypochromic, was different from the normocytic anaemia observed in *T. brucei* infected mice [29] or Nigerian mongrel dogs [30] during the acute phase of *T. b. brucei* infection. Microcytic hypochromic anaemia has previously been associated with iron deficiency [31] and could perhaps be related to failure of iron incorporation into red cell precursors or inefficient recovery of iron from the phagocytosed RBC, features which are common during acute trypanosomiasis [32]. Determination of the type of anaemia found in infected humans is complicated by presence of concurrent infectious and nutritional conditions [29]. This is compounded by the lack of appropriate haematology analysers in endemic areas, and has therefore not been systematically determined to our knowledge.

The severe progressive thrombocytopaenia reported in our study mirrors that found in other *T.b. rhodesiense* animal models [30] and human cases of sleeping sickness [28]. These findings indicate that unlike in mild cases of iron deficiency anaemia that are accompanied by thrombocytosis, the anaemia of trypanosomiasis in both humans and animals is severe and could be related to a deficit in the production of thrombopoetin [33]. Similarly, leukocyte changes are broadly consistent with findings from other non–human primate studies [3]–[4] and humans [17]. However, the strong granulocyte response that coincided with the day of first detection of trypanosomes in peripheral blood (median = 4 dpi) has not been reported before, perhaps due to the lower frequency of sampling employed in other studies. Importantly, the presence of multiple peaks of white cells during the course of the infection suggests that in spite of the widely reported immunosuppressive effects of trypanosome infections, myeloid precursor cells retain the ability to proliferate in response to dominant parasite VSG's expressed during the course of the disease. This is in agreement with findings that some bone marrow stem cells survive the damage caused by trypanosomes and retain the ability to repopulate the animal [34] and may account for the observation of very late stage leucocytosis in some individuals but not others.

The first evidence of trypanosomes in the CSF was on day 16 (range 8–40) days (Table 2). This event is recognised by WHO [6] as a definitive marker for the onset of late stage infection. The timing of CSF parasitosis was largely similar to earlier observations in the syringe passage monkey infections where the blood-brain-barrier (BBB) was breached within 7–21 days [8],[35]. Clone 3928 produced the most chronic infection of all isolates and, in monkey 554, was only detected in the CSF on day 40 after infection. Some *T. b. rhodesiense* isolates from south-eastern Africa foci and some from eastern Uganda have been reported to cause a chronic HAT infection in humans, taking relatively long to invade the CNS [24],[36]. One of the recognized markers of CNS pathology is the presence of raised numbers of leucocytes in the CSF above the background (pre-infection) levels [6], [8], 37–38. Indeed, there was positive linear association between trypanosomes in the CSF and white cell changes, suggesting that both events are primarily determined by a single cause, possibly damage to the blood-brain barrier. The numbers of trypanosomes in CSF increased dramatically as disease progressed, and clinical symptoms of disease necessitated individuals to be removed from the study on ethical grounds, marking the terminal stage.

The results of this study establish a cyclic *T. b. rhodesiense* model that more closely resembles the East African form of HAT. Although *T. b. gambiense* causes a more insidious slowly developing disease, the essential features including fever, loss of appetite, headache, fatigue, weight loss, leg paresthesis, gait difficulties and daytime somnolence are similar to symptoms observed in patients infected with *T. b. rhodesiense* [18],[39]. Thus, this disease model in which the infection is induced using the bite of a single fly can better represent the complex pathogenesis of natural HAT. This model allows more precise timing of events, such as date of infection, and the clinical and haematology features that follow. Consequently, it is hoped that the new model will gain application and facilitate studies that require good precision.
